# Molecular Imaging of Cancer Stem Cells and Their Role in Therapy Resistance

**DOI:** 10.2967/jnumed.124.267657

**Published:** 2025-01

**Authors:** Sofia N. dos Santos, Timothy H. Witney

**Affiliations:** School of Biomedical Engineering and Imaging Sciences, King’s College London, London, United Kingdom

**Keywords:** cancer stem cells, molecular imaging, PET, SPECT, therapy resistance

## Abstract

Despite recent therapeutic breakthroughs, cancer patients continue to face high recurrence and mortality rates due to treatment resistance. Cancer stem cells (CSCs), a subpopulation with self-renewal capabilities, are key drivers of refractive disease. This review explores the application of molecular imaging techniques, such as PET and SPECT, for the noninvasive detection of CSCs. By providing real-time monitoring of CSCs, these imaging methods have the potential to predict therapy resistance and guide personalized treatment approaches. Here, we cover the biological characteristics of CSCs, mechanisms of therapy resistance, and the identification and targeting of CSC-specific biomarkers with molecular imaging. Additionally, we address the challenges and opportunities for the clinical translation of CSC imaging, highlighting strategies where CSC imaging can be used to improve patient outcomes.

Recent advances in cancer care have led to declining mortality rates due to improved screening, the development of innovative therapies, and a treatment plan tailored to the individual (1). Despite these advances, patients often develop therapy resistance, resulting in recurrence. Cancer stem cells (CSCs) and drug-tolerant persister cells play a critical role in therapy resistance and tumor relapse. These cells are frequently confused because of their shared characteristics (2). However, whereas CSCs are intrinsically resistant to therapy, persister cells are defined by their transient drug-resistant state and are drug-tolerant only under therapeutic stress (2). Here, we focus on CSCs, known for their tumor-initiating and self-renewal capabilities (3). Despite evidence of their central role in cancer progression, translating these findings has stalled because of the lack of effective tools to identify and monitor CSCs in humans.

## CSC CHARACTERISTICS

CSCs were first isolated from untreated acute myeloid leukemia using CD34^+^CD38^−^ markers (4). This early pioneering research showed that only a small fraction (0.1%–1%) of tumor cells, classified as CSCs, was responsible for tumor initiation and progression. CSCs share characteristics with normal stem cells, including specific surface markers, self-renewal capacity, and unlimited proliferation (3). CSCs, however, are genetically unstable, containing mutations and epigenetic modifications that confer metabolic and phenotypic plasticity (5), allowing them to adapt their metabolism under hypoxia, evade the immune system, and support tumor growth.

## CANCER RESISTANCE MEDIATED BY CSCS

**FIGURE 1.**
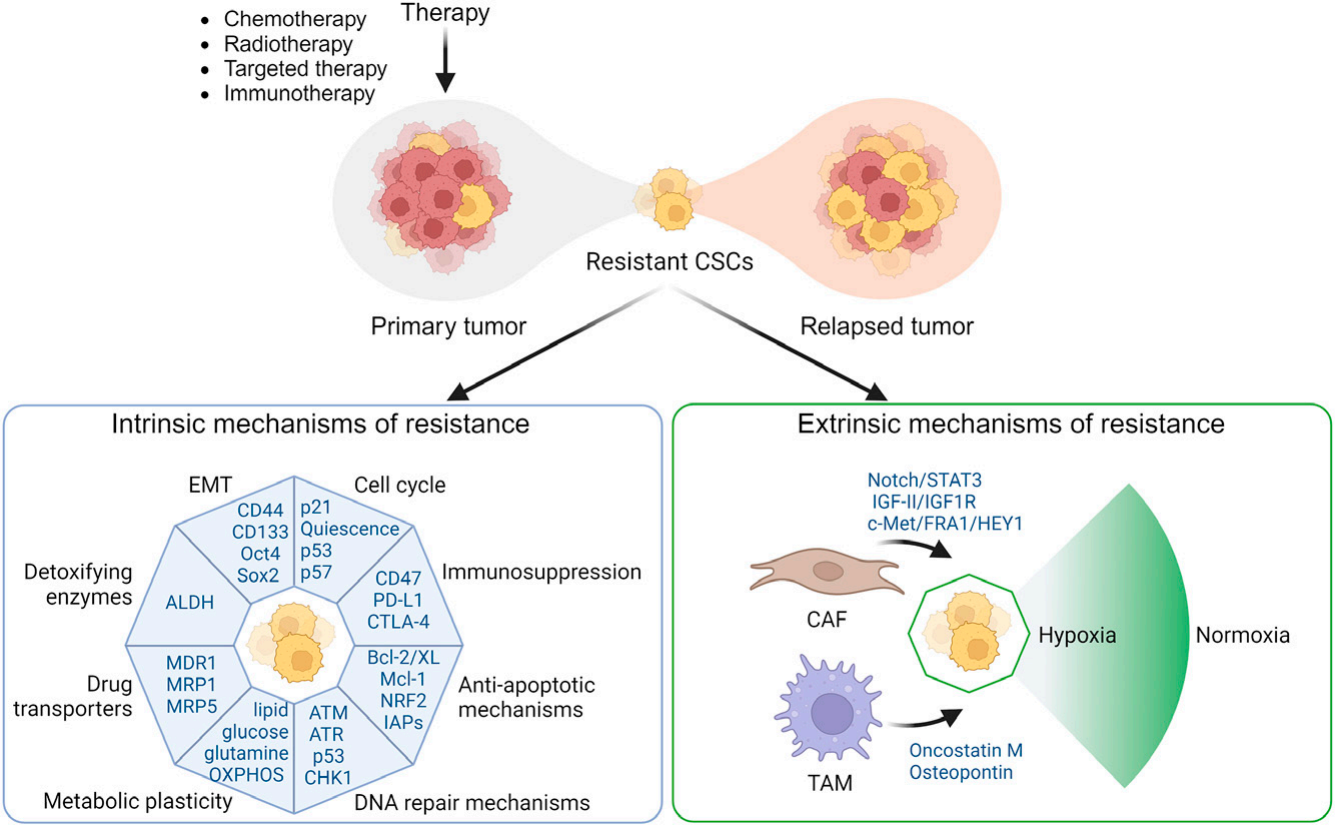
Mechanisms of therapy resistance by CSCs. Intrinsic and extrinsic mechanisms contribute to CSC-mediated therapy resistance. Intrinsic mechanisms include expression of drug transporters, detoxifying enzymes, enhanced DNA repair, immunosuppression, antiapoptotic mechanisms, epithelial–mesenchymal transition, or metabolic adaptability. Extrinsic factors include tumor microenvironment, cytokines secreted from tumor-associated macrophages and fibroblasts, and hypoxia-induced changes, which promote survival. CAF = cancer-associated fibroblasts; EMT = epithelial–mesenchymal transition; TAM = tumor-associated macrophages.

The mechanisms of CSCs in therapeutic resistance are often categorized as intrinsic or extrinsic (Fig. 1). CSCs upregulate survival pathways, which provide adaptability to therapeutic stress. Key intrinsic survival factors include…

- Overexpression of detoxifying enzymes, such as aldehyde dehydrogenase (ALDH) class 1A1 (ALDH1A1), to protect CSCs against cytotoxics (6).
- Expression of drug transporters, such as adenosine triphosphate–binding cassette transporters, P-glycoprotein, and multidrug resistance protein 1, that mediate the efflux of chemotherapies (7).
- Enhanced DNA damage response mechanisms, such as p53 mutations, enabling survival under genotoxic stress induced by chemotherapy and radiation (8).
- Evasion of apoptosis through expression of antiapoptotic proteins (B-cell lymphoma 2, B-cell lymphoma-extra-large, and the myeloid cell leukemia 1), upregulation of antioxidant pathways, and expression of inhibitors of apoptosis proteins (9).
- Progression through epithelial–mesenchymal transition driven by transcription factors such as snail, slug, zinc finger E-box binding homeobox 1, and twist-related protein 1, resulting in elevated stemness markers, such as CD44 and CD133 (10).
- Activation of quiescence, a dormant state regulated by pathways involving p53, p21, and p57, allowing CSCs to evade treatments targeting dividing cells (11).
- Metabolic plasticity dependent on oxygen availability, enabling CSCs to switch between glycolysis and oxidative phosphorylation—influenced by signals such as Wnt/β-catenin and notch pathways (12).
- Immune cell evasion by downregulating major histocompatibility complex class I to avoid cytotoxic T-lymphocyte recognition, upregulating CD47 to prevent macrophage phagocytosis, and exploiting immune checkpoints by increasing programmed death ligand 1 and cytotoxic T-lymphocyte–associated protein 4 ligands, leading to T-cell exhaustion and suppressed immune responses.

Cell-extrinsic factors in the tumor microenvironment also drive adaptive alterations in CSCs, leading to acquired therapy resistance. Tumor-associated macrophages and fibroblasts secrete cytokines that transform normal tumor cells into CSCs via TGF-β1, STAT3/Sox2, JAK2/STAT3/snail, notch/STAT3, and IGF-II/IGF1R pathways (13). Hypoxia also maintains CSC stemness via hypoxia-inducible factors, keeping cells undifferentiated, enhancing their clonogenic potential, promoting epithelial–mesenchymal transition, and increasing CSC invasiveness (14).

## MOLECULAR IMAGING OF CSCS

Given CSCs’ role in treatment failure, visualizing and monitoring CSCs could improve patient outcomes. Although frequently present in low numbers in treatment-naïve patients, CSCs are enriched after therapy (15). Consequently, whole-body imaging techniques, such as PET and SPECT, can assess CSCs in situ and may provide invaluable insights into treatment response. Recent CSC imaging strategies have targeted expression of the classic CSC biomarkers CD133, CD44, epithelial cell adhesion molecule (EpCAM), CD166, C-X-C chemokine receptor type 4 (CXCR4), and intracellular ALDH1A1. Table 1 highlights select PET and SPECT imaging agents developed over the last 5 y using a variety of approaches.

**TABLE 1.** Selected PET/SPECT Agents for CSC Imaging Developed in Last 5 Years

CSC Biomarker	Imaging agent	Targeting vector	Tumor type	Imaging modality	Stage of development	Reference
CD133	[^64^Cu]Cu-CM-2	Peptide	Hepatocellular carcinoma	PET	Preclinical	(20)
	[^89^Zr]Zr-CD133	Antibody	SCLC	PET	Preclinical	(17)
	[^89^Zr]Zr-CD133	Antibody	Colorectal	PET	Preclinical	(16)
	[^89^Zr]Zr-DFO-RW03IgG and [^89^Zr]Zr-DFO-RW03scFv-Fc	Antibody, antibody fragment	Colorectal	PET	Preclinical	(19)
	[^89^Zr]Zr-DFO-αCD133	Antibody	SCLC	PET	Preclinical	(18)
CD44	[^89^Zr]Zr-CS-GA-MLPs	Nanoparticle	Triple-negative breast cancer	PET	Preclinical	(21)
	[^89^Zr]Zr-anti-CD44 (IM7)	Antibody	Colorectal	PET	Preclinical	(22)
	[^89^Zr]Zr-scFv-Fc-CD44 [^64^Cu]Cu-scFv-Fc-CD44	Antibody fragment	Triple-negative breast cancer	PET	Preclinical	(23)
	[^89^Zr]Zr-anti-CD44v6 (U36)	Antibody	Head-and-neck squamous cell carcinoma	PET	Preclinical	(24)
CD166	[^111^In]In-DTPA-CD166tp-G18C	Peptide	Colorectal	SPECT	Preclinical	(25)
EpCAM	[^89^Zr]Zr-DFO-*N*-suc-muS110	Antibody	Melanoma	PET	Preclinical	(28)
	[^64^Cu]Cu-DOTA-PEGylated	Aptamer	Breast	PET	Preclinical	(26)
	[^99m^Tc]Tc-NB4	Nanobody	Colorectal	SPECT	Preclinical	(27)
ALDH	[^18^F]F-4b	Small molecule	Healthy animals	PET	Preclinical	(29)
	[^18^F]F-2	Small molecule	Ovarian cancer	PET	Preclinical	(30)
CXCR4	[^18^F]F-RPS-534 and [^18^F]F-RPS-547	Small molecule	Prostate cancer	PET	Preclinical	(33)
	[^99m^Tc]Tc-CXCR4-L	Peptide	Brain tumors	SPECT	Clinical	(34)
	[^99m^Tc]Tc-pentixatec	Peptide	Hematologic malignancies	SPECT	Clinical	(35)
	[^68^Ga]Ga/[^177^Lu]Lu-EPI-X4	Peptide	Leukemia	PET	Preclinical	(38)
	[^68^Ga]Ga-pentixather	Peptide	Multiple myeloma	PET	Clinical	(39)
	[^18^F]F-SFB-AMD3465	Small molecule	Breast cancer	PET	Preclinical	(40)
	[^68^Ga]Ga-pentixafor	Peptide	Various	PET	Clinical	(32)

### CD133: A Valuable Marker with Targeting Challenges

**FIGURE 2.**
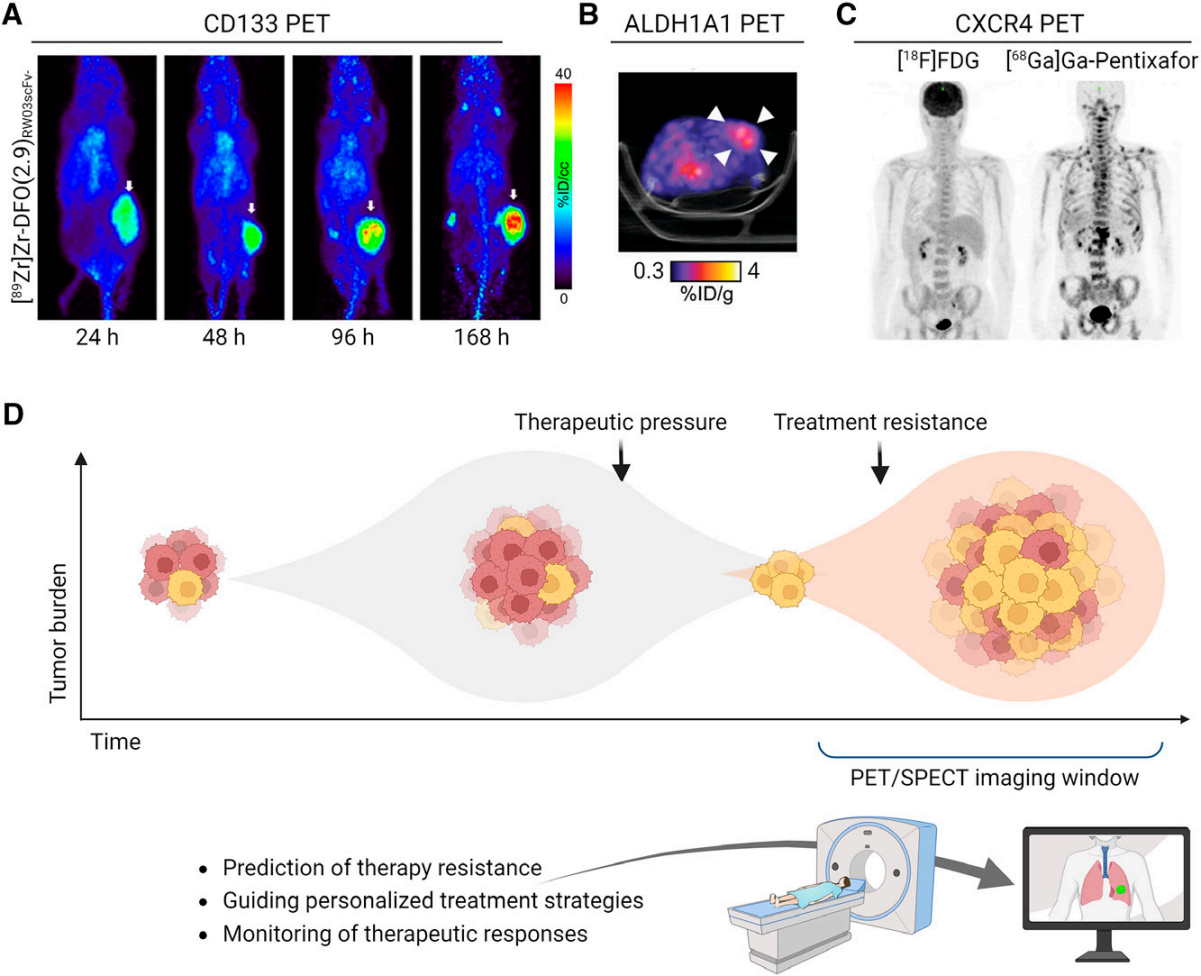
Molecular imaging of CSCs. (A) In vivo maximum-intensity projection PET images from [^89^Zr]Zr-DFO-RW03scFv-Fc in HT-29 xenograft BALB/c nu/nu mice (19). (B) Representative axial PET/CT images of [^18^F]F-2 in BALB/c nu/nu tumor-bearing mice. Arrowheads indicate tumor (30). (C) Maximum-intensity projection images showing negative lymph node uptake with [^18^F]FDG PET/CT and positive lymph node involvement with [^68^Ga]Ga-pentixafor PET/CT (37). (D) Putative CSC imaging window. CSC prevalence is low in primary tumors, increasing during treatment because of therapy-induced selection pressures, and is highest after relapse. CSC imaging during treatment may enable early detection of therapy resistance, enabling timely therapeutic adjustments. %ID = percentage injected dose.

CD133 is a cell surface glycoprotein that activates survival pathways associated with poor prognosis and therapy resistance in multiple tumor types (3). Although also expressed on normal stem cells and certain differentiated cells, CD133 is overexpressed in CSCs present in glioblastoma and small cell lung cancer (SCLC). Given its cell surface expression, most CD133-targeting imaging agents are antibody-based, paired with ^89^Zr (Table 1). One such agent was used to monitor CD133 expression in colorectal cancer models, showing that celecoxib reduced CD133 levels, which were effectively tracked by PET imaging (16). Another study detected autoantibodies against CD133 in the plasma of a SCLC patient up to a year before diagnosis (17). The study further demonstrated that [^89^Zr]Zr-labeled anti-CD133 immuno-PET could identify SCLC tissue in mice, suggesting a role for CD133 imaging in early cancer detection. Building on this, Sarrett et al. (18) developed a [^89^Zr]Zr-labeled probe for PET imaging and a [^177^Lu]Lu-labeled therapeutic, which provided a survival benefit in SCLC models. Researchers have also used antibody fragments and peptides to address the limitations of antibody-based imaging, such as slow pharmacokinetics and limited tissue penetration. A humanized antibody fragment, [^89^Zr]Zr-DFO-RW03_scFv-Fc_, that has been developed has improved tumor uptake and reduced off-target binding compared with the full antibody (Fig. 2A) (19). Similarly, Hu et al. (20) created a [^64^Cu]Cu-labeled peptide PET tracer to track CD133 in liver cancer. CD133 remains a complex biomarker for CSC imaging because of variable expression across cancers and within tumors. CD133 glycosylation also hinders antibody binding, and tumors can be initiated by both CD133^+^ and CD133^−^ cells, meaning it is not a true CSC biomarker. Nonetheless, its association with therapy resistance and poor prognosis in glioblastoma and SCLC makes it valuable for outcome prediction.

### CD44: A Versatile but Complex Target

CD44 is a transmembrane glycoprotein involved in cell adhesion, migration, and progression in multiple indications, including triple-negative breast cancer and gastric, colorectal, and head and neck squamous cell carcinoma (3). As a CSC marker, CD44 imaging is complicated by its alternative splicing and glycosylation, which affect target binding. Variants CD44v6 and CD44v9 are associated with tumor aggressiveness and the CSC phenotype, with isoform-specific imaging a potential strategy to improve CSC specificity. Recent advances include the development of a [^89^Zr]Zr-labeled nanoliposome with chitosan for CD44 targeting, which increased tracer stability, reduced premature clearance, and offered potential as a radiotheranostic (21). Another approach used a [^89^Zr]Zr-anti-CD44 antibody targeting human and murine isoforms in CD44-positive colon cancer cells (22). Diebolder et al. (23) developed a bivalent scFv-Fc antibody fragment radiolabeled with [^89^Zr]Zr and [^64^Cu]Cu, showing good specificity and retention in CD44-positive breast cancer models. However, late imaging time points were required. To overcome these issues, pretargeted immuno-PET approaches have been explored using a chimeric anti-CD44v6 antibody and a [^89^Zr]Zr-labeled tetrazine, which reduced radiation exposure while maintaining favorable imaging characteristics (24). These findings highlight the need for a refined approach for CD44 imaging, emphasizing the requirement for isoform-specific targeting to enhance CSC specificity, which will better inform the selection of CD44 therapies.

### CD166: An Emerging Target

CD166 is involved in cell adhesion and has been identified as a CSC marker in colorectal, ovarian, and head and neck squamous cell carcinoma cancers. Its upregulation is associated with hypoxia, epithelial–mesenchymal transition, and treatment resistance, making it a critical marker to identify resilient CSCs (3). Few CD166-based imaging agents exist, possibly because CD166 is expressed in both normal and cancerous tissues. Despite these challenges, [^111^In]In-DTPA-CD166tp-G18C has shown promise for detecting CD166^+^ CSCs in a mouse model of colorectal cancer (25). A better understanding of CD166 function in CSCs, however, is needed to harness its potential in imaging.

### EpCAM: A Marker for Epithelial Cancers

EpCAM is significantly overexpressed, often by 1 to 2 orders of magnitude, in epithelial cancers, such as gastric, colon, and breast. Traditionally known for its role in cell–cell adhesion within epithelial tissues, EpCAM facilitates oncogene transcription and epithelial–mesenchymal transition, which are crucial to maintain the CSC phenotype (3). Recent developments include a [^64^Cu]Cu-DOTA-PEGylated aptamer (26), which binds EpCAM-expressing CSCs in breast cancer models. Despite superior tissue penetration and faster clearance than antibodies, challenges such as high liver uptake and low tumor retention remain. Similarly, [^99m^Tc]Tc-NB4 nanobodies targeting EpCAM^+^ CSCs with high specificity were used to image colorectal cancer by SPECT (27). However, low tumor accumulation may limit its utility. Bispecific antibodies targeting EpCAM, such as bispecific T-cell engager molecules, may provide an alternative strategy (28). Despite the promise of the bispecific antibodies, their biodistribution was predominantly influenced by the CD3-targeting arm rather than the EpCAM-targeting arm. The broad expression of EpCAM across both normal epithelial tissues and tumors further poses a challenge for CSC-specific imaging, suggesting that combining EpCAM with other CSC markers may be a more effective strategy.

### ALDH: Targeting the Metabolic Activity of CSCs

ALDH is an intracellular enzyme that oxidizes aldehydes to carboxylic acids. High activity of ALDH is a hallmark of CSCs and is associated with poor prognosis and resistance to chemotherapy across various cancers, including breast, lung, and ovarian (3). To date, assessment of ALDH activity has relied on fluorescence-based assays, which are effective in isolated cells but have limited use in solid tumors. Our group has developed small-molecule–based aldehyde probes that become trapped in CSCs with high ALDH1A1, providing a functional readout of enzymatic activity (29). Blood oxidation of the lead radiotracer, [^18^F]F-4b, however, limited its in vivo application. To prevent premature oxidation, we developed an esterase-cleavable prodrug radiotracer to protect the aldehyde ([^18^F]F-2), which was effectively retained in drug-resistant ovarian cancer cells with high ALDH1A1 activity (30). Despite generating clear tumor-to-background images in vivo (Fig. 2B), the radiotracer could not distinguish tumors with varying levels of ALDH1A1, primarily because of premature liberation of the aldehyde-protecting group. We are currently developing small-molecule theranostics for the imaging and treatment of ALDH1A1-positive tumors (WO2024100095A1). Alternative strategies that image the concentration of intracellular aldehydes through [^18^F]F-NA_3_BF_3_-aldehyde complex formation have also been developed (31) and could complement the readout on ALDH activity.

### CXCR4: A Multifaceted Target with Evolving Imaging and Therapeutic Strategies

CXCR4, a chemokine receptor, plays a pivotal role in cancer progression, metastasis, and CSC maintenance through signaling pathways such as PI3K/AKT and JAK/STAT. Despite CXCR4’s broad expression profile, its overexpression in certain cancers is attributed to CSCs and is linked to poor outcomes (3). Recently, tracer development has focused on peptides and small molecules, offering faster clearance and broader applicability than antibody-based approaches. Among these agents, [^68^Ga]Ga-pentixafor (32) stands out, having been safely used in over 1,000 patients to image CXCR4 expression in multiple cancers. Serving as a benchmark in CXCR4-targeted imaging (Fig. 2C), its therapeutic counterpart, pentixather, labeled with [^177^Lu]Lu or [^90^Y]Y, has shown promise for the treatment of refractory multiple myeloma. Despite the success of [^68^Ga]pentixafor, researchers are exploring alternatives with improved pharmacokinetics, enhanced affinity, or reduced off-target binding (Table 1). The introduction of [^18^F]F-labeled small-molecule tracers, such as [^18^F]F-RPS-534, has improved tumor uptake and background contrast while using a radionuclide with superior imaging characteristics (33). Newer agents, such as [^99m^Tc]Tc-pentixatec, offer clinical advantages due to their compatibility with SPECT, a more accessible and cost-effective imaging modality (34*,*^35^). CXCR4 is increasingly recognized not only for tumor visualization but also for its ability to assess tumor heterogeneity and resistance mechanisms—particularly in CSC-rich cancers. Blocking CXCR4 disrupts metastatic pathways and reverses immunosuppression, further highlighting the importance of this CSC biomarker.

## CLINICAL TRANSLATION: CHALLENGES AND OPPORTUNITIES

Translating preclinical findings in CSC imaging into clinical practice presents several challenges. One significant hurdle is the heterogeneity of CSCs within and across tumors, making it difficult to develop universally applicable imaging agents. A key opportunity might lie in the identification and targeting of CSC biomarkers upregulated in therapy-resistant cancers (Fig. 2D).

### Timing and Value of CSC Imaging

Understanding the prevalence of CSCs at different tumor stages is crucial for effective imaging strategies. Typically, CSCs are present in low frequencies in primary tumors, often accounting for less than 1% of the tumor cell population (36). For instance, CSCs constitute about 2% of the tumor cell population in breast cancer and about 0.5%–1% in colorectal cancer. In more aggressive cancers such as triple-negative breast cancer, CSCs can exceed 10% of the tumor cell population. Their proportion, however, significantly increases after relapse because conventional treatments selectively eliminate differentiated tumor cells, leaving behind the therapy-resistant CSCs. Imaging the increased prevalence of CSCs on tumor relapse is therefore feasible and holds great prognostic value. Detecting CSCs in real time as resistance emerges during treatment may also provide a larger window for intervention than is currently possible. Through dose escalation or the initiation of second-line therapy, patient outcomes can potentially be improved. Longitudinal imaging studies tracking CSC dynamics during treatment and remission could also provide critical insights into resistance mechanisms and help identify predictive biomarkers.

### CSC Biomarkers

Accurately identifying CSCs remains a significant challenge because of the lack of specific biomarkers. Current biomarkers are also expressed in normal stem cells and non-CSC tumor cells, complicating image interpretation and delaying the development of anti-CSC radionuclide therapies. To overcome these challenges, a multifaceted approach is necessary involving, first, development of novel radiopharmaceuticals that target known CSC markers with enhanced specificity and improved pharmacokinetics; second, optimization of clinical impact, focusing imaging efforts on specific cancer subsets such as therapy-resistant or aggressive tumors (e.g., targeting CD133 in glioblastoma or CD44 in triple-negative breast cancer, both of which are associated with CSCs in these indications); third, prioritization of the identification and validation of new biomarkers with improved CSC specificity that are uniquely upregulated in therapy-resistant cancers, such as ALDH isoforms; and fourth, exploration of interactions between CSCs and their microenvironment, including stromal and immune cells, to reveal novel imaging targets and strategies.

## CONCLUSION

Advanced molecular imaging techniques such as PET and SPECT provide a real-time, noninvasive understanding of CSC behavior and prevalence. Through the development of novel tracers that target new CSC-specific biomarkers, we can improve the detection and monitoring of CSCs. Imaging CSCs during treatment holds substantial clinical value by facilitating the early detection of resistance, allowing for timely therapeutic adjustments. The increased frequency of CSCs in relapsed tumors also emphasizes the need to develop therapies that specifically target these cells. Combining these novel therapies with advanced CSC imaging techniques in the clinic will improve the management and understanding of therapy-resistant cancers, ultimately improving cancer treatment outcomes and patient survival rates.

## DISCLOSURE

This study was funded through a Wellcome Trust Senior Research Fellowship (220,221/Z/20/Z) to Timothy Witney, who is an inventor on a patent for ALDH1A1 imaging (WO2024100095A1). Both of the authors are founders and shareholders of Nuclide Therapeutics Ltd., a radionuclide company that develops radiotheranostics targeting ALDH1A1. No other potential conflict of interest relevant to this article was reported.
